# Effect of Nanosilica on Mechanical Properties and Microstructure of PVA Fiber-Reinforced Geopolymer Composite (PVA-FRGC)

**DOI:** 10.3390/ma12213624

**Published:** 2019-11-04

**Authors:** Hasan Assaedi, Thamer Alomayri, Ayesha Siddika, Faiz Shaikh, Hatem Alamri, Subaer Subaer, It-Meng Low

**Affiliations:** 1 Department of Physics, University College in AlJumum, Umm Al-Qura University, P.O. Box 715, Makkah 21955, Saudi Arabia; hsassaedi@uqu.edu.sa (H.A.); halamri@uqu.edu.sa (H.A.); 2 Department of Physics, Faculty of Applied Science, Umm Al-Qura University, P.O. Box 715, Makkah 21955, Saudi Arabia; tsomayri@uqu.edu.sa; 3 Department of Civil Engineering, Pabna University of Science & Technology, Pabna 6600, Bangladesh; ayeshace@pust.ac.bd; 4 Department of Civil Engineering, Curtin University, GPO Box U1987, Perth 6845, Australia; s.ahmed@curtin.edu.au; 5 Department of Physics, Faculty of Mathematics and Natural Sciences, State University of Makassar, Jl. Daeng Tata Raya, Makassar 90224, Indonesia; subaer@gmail.com; 6 Department of Physics and Astronomy, Curtin University, GPO Box U1987, Perth 6845, Australia

**Keywords:** geopolymer, nanosilica, PVA fibers, mechanical properties

## Abstract

This paper presents the effects of various nanosilica (NS) contents on the mechanical properties of polyvinyl alcohol (PVA) fiber-reinforced geopolymer composites (PVA-FRGC). Microstructure analysis with X-ray diffraction (XRD) and scanning electron microscopy (SEM) was used to characterize the geopolymer composites. The results showed that the mechanical properties in terms of compressive strength, impact strength, and flexural behavior were improved due to the addition of NS to the PVA-FRGC. The optimum NS content was 1.0 to 2.0 wt%, which exhibited highest improvement in the above mechanical properties. Microstructure analysis showed that the addition of NS up to an optimum level densified the microstructure of the matrix as well as the PVA fiber–geopolymer matrix interface.

## 1. Introduction

Carbon dioxide emission during manufacturing of ordinary Portland cement is one of the major environmental pollution issues worldwide. “Geopolymer” is a terminology coined by Davidovits [1] for a sustainable and alternative binder that is produced by activating alumina–silicate source material using alkali solutions. The use of geopolymer binders can reduce around 80–90% of carbon dioxide emissions [1]. Significant research has been conducted to study the various properties of geopolymers, and they show that most of the properties are superior or comparable to their counterpart cement-based binders. However, despite the above improvements, the brittleness of geopolymers is still similar to cement concrete. To address this limitation, researchers have studied the use of various types of short, randomly distributed fibers to reinforce geopolymers and improve their tensile and flexural behavior. A number of studies have reported the use of cotton and flax fibers [2,3,4], PVA fiber [5,6], polyethylene fiber (PE) [7,8], glass fiber [9,10], carbon fiber [11], and steel fiber [5,12] to reinforce geopolymers. In all the above studies, an improvement in the flexural and tensile strength of geopolymer composites was reported due to the addition of fibers.

Nanotechnology and the use of nanomaterials are recently being applied in concrete and cement-based composites to improve their properties by manipulating the microstructure of these materials at nano- and microscale. Significant improvement in the properties of concrete- and cement-based composites has been reported due to the addition of various nanomaterials [13,14,15,16]. Nanomaterials have also been shown to make a significant improvement in the bond of steel fiber with cement matrix in concrete [17]. Nanomaterials are also added in geopolymers to improve their properties. Due to their pore-filling effect and chemical reaction with geopolymer gels, the matrix of geopolymers is densified, leading to improved mechanical and durability properties. However, not enough studies have reported on the effect of nanomaterials on the properties of fiber-reinforced geopolymers.

A geopolymer matrix with reinforcing short fiber and nanomaterials can help achieve enhanced mechanical properties of composites without significant increase in cost and environmental degradation. Scientific research in this area is beneficial for innovation and advanced material development. The current study focuses on the effect of various nanosilica (NS) additions to a geopolymer matrix reinforced using PVA fibers. Previous research has alluded that the addition of PVA fibers in geopolymer matrix increases the compressive strength of composites by up to 40–70% [18] depending on the amount of PVA fiber. The literature further indicates that the use of PVA fibers is as high as 7%, which is reasonably very high and might impart significant cost to the composite. In addition, the mixing and uniform dispersion of 7% PVA fiber in concrete is very challenging and often results in nonuniform dispersion of fiber and fiber balling. As a result, the composites exhibit excessively high volume of pores and voids, which adversely affect the mechanical and durability properties of the composite. Recent studies have shown that the addition of 2 vol% of PVA fiber exhibits a significant improvement in the mechanical properties of cement and geopolymer composites, leading to strain hardening behavior with enhanced ductility [19,20]. In previous studies [18,19,20,21], researchers have also used 6 wt% of nanosilica in their PVA fiber-reinforced geopolymer composites (PVA-FRGC). The addition of such high amounts of NS in geopolymer composites is not cost-effective and might also cause nonuniform dispersion due to agglomeration of nanomaterials in water. Due to significantly higher surface area and nanosize of their particles, nanomaterials often agglomerate in the presence of water if proper mixing (e.g., ultrasonication) method is not adopted. Hence, in this study, 2 vol% of PVA fiber was used to reinforce geopolymer composites with various NS contents of 1.0 wt% to 3.0 wt% to identify the optimum NS content. Various mechanical properties of PVA-FRGC were studied, and the microstructure of PVA-FRGC after mechanical testing was examined to evaluate the effect of NS in improving the geopolymer matrix as well as the PVA–geopolymer matrix interface.

## 2. Experimental Procedure

### 2.1. Materials

**Figure 1:**
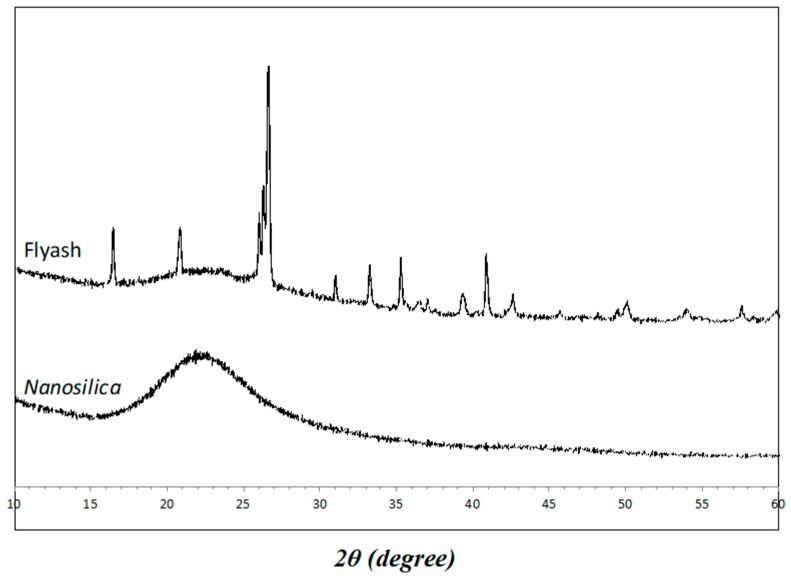
XRD patterns of fly ash and nanosilica particles.

Low-calcium fly ash meeting ASTM Class F (obtained from Eraring power station, NSW, Australia) was used as the source material for geopolymers. The chemical composition of the fly ash used in this study is shown in Table 1. Nanosilica was supplied by Nanostructured and Amorphous Materials, Inc. (Katy, TX, USA), and its standard diameter was 50 nm. For geopolymerization, the alkaline activator employed was a mixture of sodium hydroxide (NaOH) and sodium silicate (Na_2_SiO_3_) solutions. The sodium hydroxide flakes were 98% pure. Sodium silicate solution contained 29.4% SiO_2_, 14.7% Na_2_O, and 55.9% water by mass. The PVA fiber was supplied by Kuraray Co., Ltd. of Japan (Tokyo). The properties of PVA fibers are shown in Table 2. The XRD spectra of NS and fly ash are presented in Figure 1. The crystalline phases were indexed using powder diffraction files (PDFs) provided by the Inorganic Crystal Structure Database (ICSD). The diffraction pattern of the NS powder exhibited a complete amorphous (glass) phase, whereas the fly ash showed two main crystalline phases: quartz and mullite.

### 2.2. Preparation of PVA–Geopolymer Nanocomposites

In this study, four series of PVA-FRGC composites were considered. The first series was control PVA-FRGC containing no NS and 2 vol% PVA fiber. This series was denoted as PVA-FRGC-0. In second, third, and fourth series, 1.0 wt%, 2.0 wt%, and 3.0 wt%. NS was added and termed as PVA-FRGC1, PVA-FRGC-2, and PVA-FRGC-3, respectively. In all four composites, a constant alkaline solution to fly ash ratio of 0.45 was employed, and the ratio of the Na_2_SiO_3_/NaOH solution was fixed at 2.5. The molarity of the NaOH solution was 10 M, and it was mixed with Na_2_SiO_3_ solution to prepare the alkali solution. The fly ash and NS was first dry mixed for 3 min followed by the addition of alkali solution and further mixing of 3 min. The PVA fibers were then added and mixed for a further 3 min. The freshly mixed composites were then poured into the moulds, which were then vibrated in a vibrating table to remove any entrapped air. The moulds containing freshly mixed composites were covered by plastic sheet to prevent any moisture loss and were kept for 24 h at 80 °C in an oven. The curing under ambient temperature condition was continued for 28 days. Table 3 shows the mix designation and mix proportions of each series.

### 2.3. Microstructure Characterisation

A scanning electron microscope (SEM; Zeiss EVO-40, Carl Zeiss, Oberkochen, Germany) was used to capture the microstructure images of the fracture surfaces of the specimens. In each series, a small portion of the fractured specimen was cut using a diamond cutter and was placed in a vacuum desiccator. This was necessary for gassing-out before subsequent display on aluminum stubs. They were then coated with platinum to prevent charging during the examination. X-ray diffraction patterns were collected on a D8 Advance diffractometer (Bruker AXS, Karlsruha, Germany) using a Cu Kα source. The data was accumulated using a nominal 2θ step size of 0.01°, a count time of 0.5 s per step, and a 2θ range of 10°–70°.

### 2.4. Mechanical Properties

Eighteen specimens were cast in each series to measure the compressive, flexural, and impact strengths of the composites. Compression test was performed according to ASTM C 109 on 20 mm cube specimens. Flexural test was performed according to ASTM D790-10. Three-point bend tests of prism specimens (60 mm × 20 mm × 20 mm) with displacement control of 1 mm/min were conducted. The following equation was used to determine the flexural strength (*σ*_F_):

(1) $$\sigma_{F}=\frac{3}{2}\frac{P_{m}S}{WD^{2}}$$

where *P_m_* is the maximum load, *S* is the span, *W* is the width, and *D* is the thickness of the specimen.

Indicators of toughness indices (*I_5_* and *I_failure_*) were assessed as they characterize flexural toughness of geopolymer composites. Using the initial slope of the load–displacement curve obtained in the bending test, the flexural modulus was computed as follows:

(2) $$E_{F}=\frac{S^{3}}{4WD^{3}}\left(\frac{\Delta P}{\Delta X}\right)$$

where $\frac{\Delta P}{\Delta X}$ is the slope of the load deflection curve.

For impact strength investigation, a Zwick Charpy with a 1.0 J pendulum hammer was employed on five rods of 60 mm length. Calculations of the impact strength (*σ_i_*) were estimated applying the following equation:

(3) σi=EA

## 3. Results and Discussion

### 3.1. Mechanical Properties

#### 3.1.1. Compressive Strength

**Figure 2:**
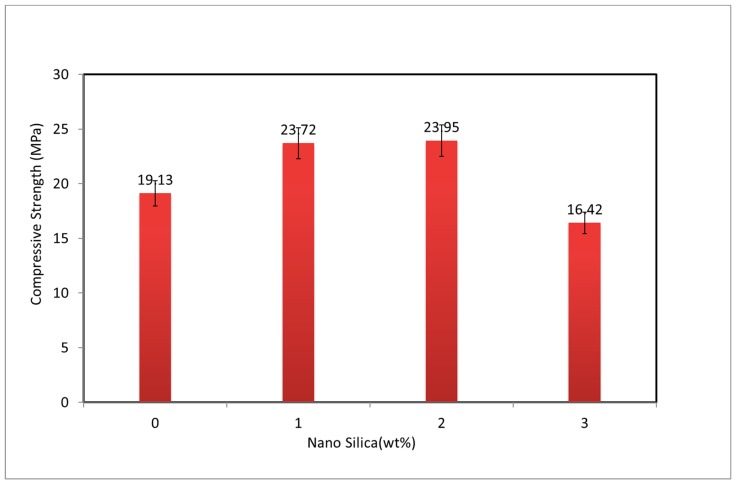
Compressive strength of PVA fiber-reinforced geopolymer composites (PVA-FRGC) at various nanosilica contents.

The effect of various NS contents on the compressive strength of PVA fiber-reinforced geopolymer composite is shown in Figure 2. Standard deviation of the results in terms of error bars are also shown in the same figure. The results presented are average of six specimens. Compared to the composite without NS, the addition of 1.0 and 2.0 wt% NS improved the compression strength by about 24–25%. However, at NS content of 3.0 wt%, the compressive strength of PVA-FRGC was lower than the control PVA-FRGC. This could be attributed to the agglomeration of NS during wet mixing due to its higher content. The observed result is consistent with other reported results on geopolymers containing nanosilica [13,14,15]. The optimum content of NS was found to be 2.0 wt% in terms of maximum compressive strength of the composites, which was 25.0% higher than the control composite, as shown in Figure 1. High ratio of surface area to volume, high dissolution rate, and low binding energy led to accelerated geopolymerization process due to the addition of NS, which strongly influenced the compressive strength of the PVA-FRGC. Therefore, the addition of amorphous nanosilica produced more geopolymeric product, which led to improvement in load carrying capacity. Nanosilica addition also results in filler effect within the geopolymer matrix and makes denser composites [13], which was seen in nanosilica contents up to 2.0 wt%. However, further increase in NS caused loss in strength. This trend echoes current research outcomes on geopolymers with NS addition [14,15]. This is evident in the work of Gao et al. [17], where the porosity of geopolymer composites decreased with the addition of NS up to a certain limit, but any further increase, e.g., 3.0 wt% nanosilica, exhibited the opposite phenomenon.

#### 3.1.2. Flexural Strength and Flexural Modulus

**Figure 3:**
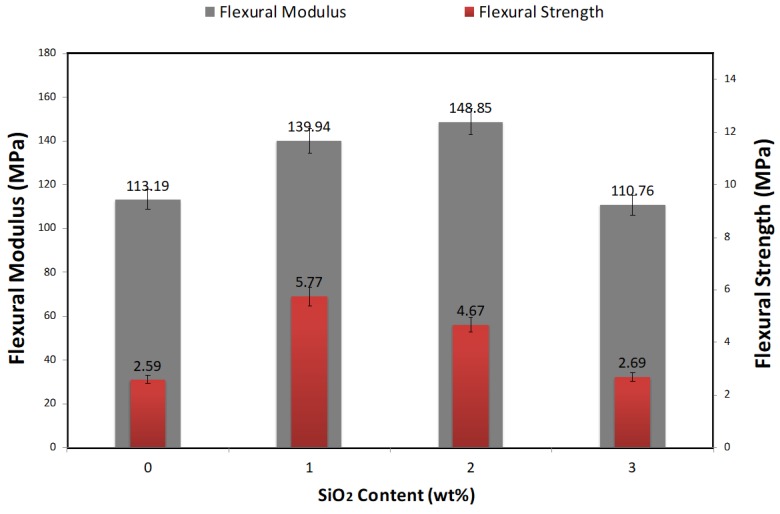
Variation of flexural modulus and flexural strength of all composites as a function of nanosilica contents.

High resistance to deformation under bending action is referred to as flexural modulus. Bending test results provided noteworthy information about the PVA-FRGC with and without NS particles. To improve the tensile and flexural properties and cracking resistance of geopolymer matrix, short PVA fibers and NS can be used together. High flexural modulus is the sign of higher strain capacity, which prevents major cracking. When 1.0 and 2.0 wt% of NS are added in PVA-FRGC, the flexural modulus was increased by 23.6% and 31.5%, respectively, as shown in Figure 3. The uniformly distributed PVA fibers and the compact microstructure of the geopolymer matrix due to NS addition improved the fiber–geopolymer interface bond, caused perfect stress distribution within the matrix, and enhanced the strain of the composite specimen under peak load. Good internal adhesion between the PVA fibers and NS-enriched dense geopolymer matrix enhanced the bond strength of PVA fibers in the composite, thereby bridging the microcracks and improving the deflection capacity. The flexural modulus of PVA-FRGC was improved using NS, and the optimum quantity for NS was 2.0 wt%. In comparison to the control specimen, flexural strength that was 2 times higher was obtained for PCA-FRGC by adding 1.0 wt% NS. Such a perfect combination obtained from geopolymerization activity and filler effect of NS with crack bridging mechanisms of PVA enhanced the flexural strength of geopolymer specimens up to 122.7%. Adding NS beyond its optimum limit, however, reduced the flexural strength. For instance, in this study, about 53.7% lower flexural strength was observed when 3.0 wt% NS was used in PVA-FRGC instead of 1.0 wt% NS.

**Figure 4:**
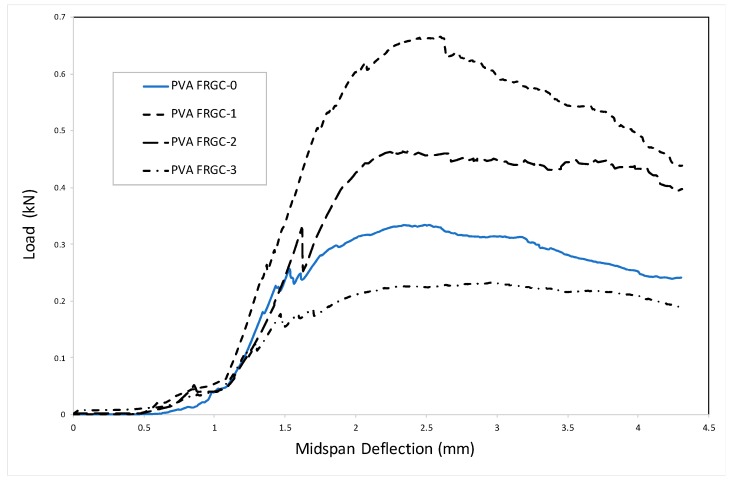
Load–midspan deflection of all composites.

**Figure 5:**
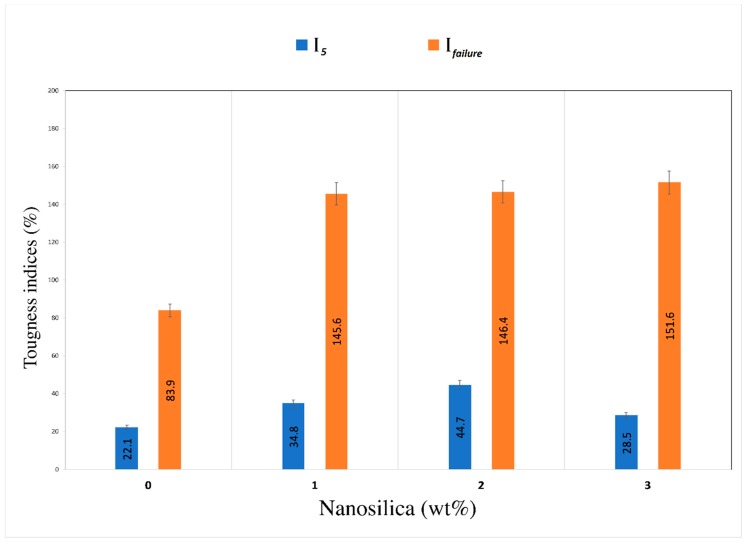
Toughness indices of composites.

By studying the composite toughness, the previous findings on this could be ascertained. The load–midspan deflection for all composites is shown in Figure 4. The toughness index *I_5_*, by definition, is the ratio between the area of triple the initial fracture deflection to the initial fracture deflection, whilst *I_failure_* can be calculated at 4.5 mm deflection for every specimen toughened with PVA fibers. All composites displayed proper stiffness because of the capacity of PVA to bridge the cracks and bear greater load, which overcame the issue of brittleness and sudden failure of the geopolymer. However, the addition of nanosilica particles showed remarkable variations on the results of flexural toughness. Figure 5 shows the results of toughness indices as calculated from the load–midspan deflection curves of all composites. Composites containing 1.0, 2.0, and 3.0 wt% NS displayed enhancement of 50–100% in *I_5_* toughness indices and 70–80% in the *I_failure_* toughness indices compared to the PVA-FRGC without nanosilica. Amongst composites with various quantities of NS, the composite with 1.0 wt% was found to display the maximum strength. However, the load–midspan deflection curve showed higher rate of load reduction with an increase in deflection beyond the peak load of 660 kN, which may be attributed to the fiber fracture effect. In contrast, composites with 2.0 wt% exhibited lower strength but slower reduction in load beyond the peak load point. This may be due to the fiber pull-out effect as the adhesion between the geopolymer matrix and PVA fibers was lower compared to the composite with 1.0 wt% nanosilica. It seems from the above results that, at nanosilica content of 1.0 wt%, its dispersion was much better than the other two nanosilica contents, resulting in higher flexural behavior of the PVA fiber-reinforced geopolymer composite.

#### 3.1.3. Impact Strength

**Figure 6:**
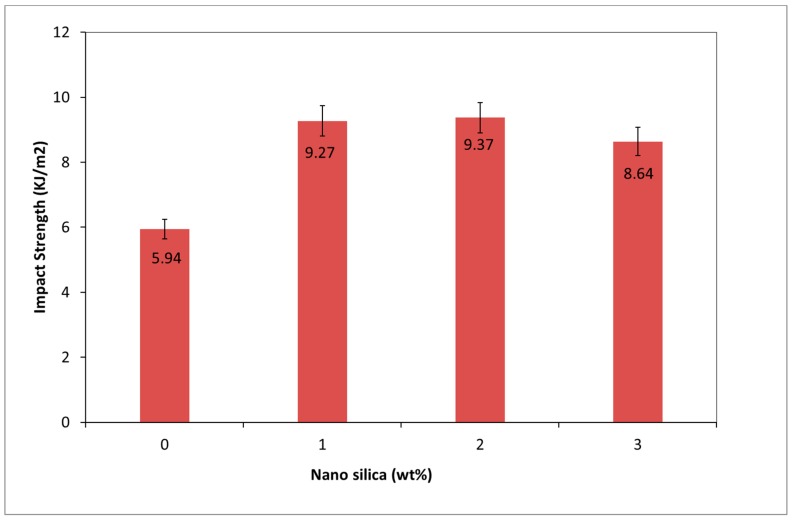
Variation of impact strength of geopolymer composites as a function of nanosilica content.

The aptitude of the matter to endure force is referred to as the impact strength. The effect of NS addition on impact strength possessed by PVA-FRGC is shown in Figure 6. It can clearly be seen that the presence of NS significantly improved the impact strength of PVA-FRGC beyond the optimum content. The impact strength of PVA-FRGC containing 1.0 and 2.0 wt% NS was increased by about 57–58% compared to PVA-FRGC without NS. The addition of 3.0 wt% NS in the composite, on the other hand, deviated this trend slightly. This reduction in impact strength was linked to the poor dispersion of NS into the geopolymer matrix due to agglomeration. This led to poor bond between the matrix and the PVA fibers.

### 3.2. Microstructural Analysis

**Figure 7:**
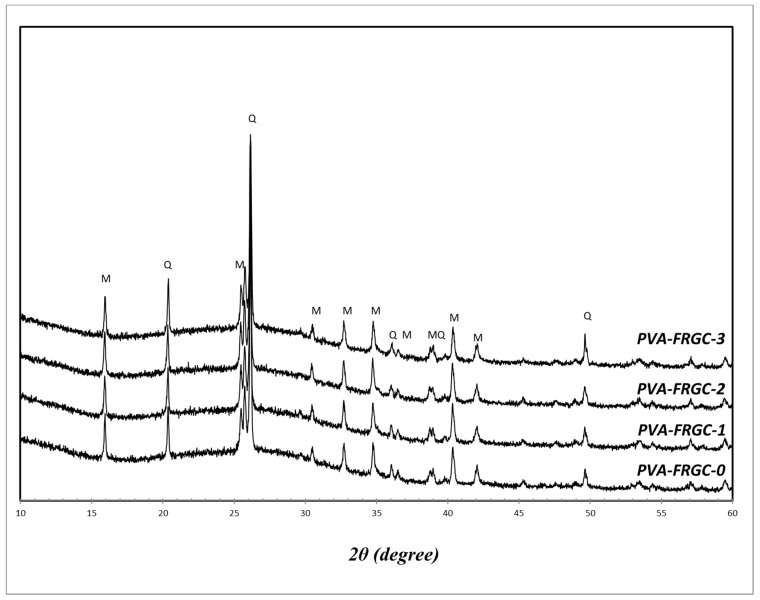
XRD patterns of geopolymer composites.

Figure 7 shows the XRD patterns of PVA-FRGC containing various NS contents as well as control PVA-FRGC. It can be seen that the control composite had the same crystalline phases as fly ash, so those phases were not reactive during the geopolymer reaction, and their role was limited to being a filler in the geopolymer matrices. However, the amorphous hump ranging between 2θ angle of 15° and 30° characterized the reactive aluminosilicate content in alkaline solution through the reaction. The composites containing the nanosilica had similar pattern to that of the control composite, and no apparent differences could be observed. This was due to the small proportion and the amorphous nature of the added nanosilica particles, which is believed to have participated in the geopolymer gel formation.

**Figure 8:**
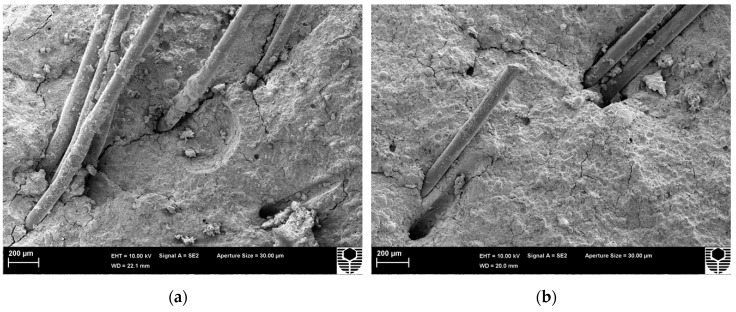

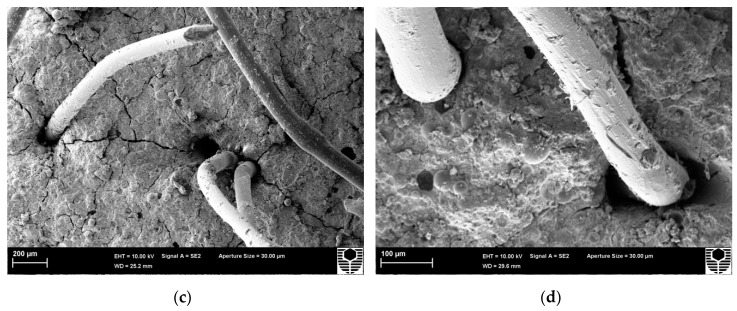
SEM micrograph showing the microstructure of fractured surface of (**a**) PVA-FRGC-1, (**b**) PVA-FRGC-2, (**c**) PVA-FRGC-3 and (**d**) close-up of PVA-FRGC-1.

The microstructural analyses of the broken specimen surfaces of PVA-FRGC-1, PVA-FRGC-2, and PVA-FRGC-3 are shown in Figure 8. Slight variations can be seen on the structure of the samples. The composite containing 1.0 and 2.0 wt% NS showed denser microstructures of the matrix, leading to strong bond with PVA fibers, as shown in Figure 8a,b. However, the composite with 3.0 wt% NS appeared relatively less dense and had more cracks, which might have resulted in poor bonding with the fibers, as shown in Figure 8c. Close-up view of PVA-FRGC-1 (Figure 8d) showed some scratches of the PVA fiber surfaces, indicating higher frictional resistance during debonding and pull-out of fibers due to the denser microstructure of the geopolymer containing 1.0 wt% NS than others. The better mechanical properties of the composite containing 1.0 wt% NS also agreed well with this observation.

## 4. Conclusions

In this research, the mechanical properties of PVA fiber-reinforced geopolymer composites containing different amounts of nanosilica were evaluated. The suitable content of nanosilica was established as 1.0 to 2.0 wt%. The PVA fiber-reinforced geopolymer nanocomposites containing 1.0 and 2.0 wt% nanosilica improved the compressive strength, flexural strength, and impact strength in comparison to the PVA fiber-reinforced geopolymer composite without nanosilica. However, increasing the nanosilica content beyond 2.0 wt% negatively affected the mechanical properties of the composites. SEM micrographs of PVA fiber-reinforced geopolymer composite containing 1.0 and 2.0 wt% nanosilica showed denser microstructure of the geopolymer matrix than that containing 3.0 wt% nanosilica.

## Figures and Tables

**Table 1:** Chemical composition of fly ash (unit: wt%).

SiO_2_	Al_2_O_3_	CaO	Fe_2_O_3_	K_2_O	MgO	Na_2_O	P_2_O_5_	SO_3_	TiO_2_	MnO	BaO	LOI
63.13	24.88	2.58	3.07	2.01	0.61	0.71	0.17	0.18	0.96	0.05	0.07	1.45

**Table 2:** Properties of polyvinyl alcohol (PVA) fibers.

Length (mm)	Diameter (mm)	Modulus of Elasticity (MPa)	Fiber Strength (MPa)	Density (gm/cm^3^)	Elongation (%)
8	0.04	40000	1600	1.3	6

**Table 3:** Mix proportions of composites.

Series	NS (wt%)	Fly Ash (g)	NaOH (g)	Na_2_SiO_3_ (g)	NS (g)	PVA (%)
PVA-FRGC-0	0	1000	214.5	535.5	0	2.0
PVA-FRGC-1	1	1000	214.5	535.5	10	2.0
PVA-FRGC-2	2	1000	214.5	535.5	20	2.0
PVA-FRGC-3	3	1000	214.5	535.5	30	2.0
